# How to better select SARS‐CoV‐2 preservation solution of virus nucleic acid testing

**DOI:** 10.1002/jcla.24956

**Published:** 2023-09-03

**Authors:** Xiuzhi Duan, Letao Huang, Xuchu Wang, Ying Ping, Pan Yu, Weiwei Liu, Yiyi Xie, Zhihua Tao

**Affiliations:** ^1^ Department of Laboratory Medicine The Second Affiliated Hospital of Zhejiang University School of Medicine Hangzhou China; ^2^ School of Public Health Xiamen University Xiamen China

**Keywords:** guanidine salt, nucleic acid testing, SARS‐CoV‐2, virus preservation solution

## Abstract

**Background:**

Sampling and testing for SARS‐CoV‐2 is a widely recognized method for identifying patients with COVID‐19. However, there is limited research available on the stability of nucleic acids in viral storage solutions.

**Methods:**

This paper investigates the components that provide better protection for virus and nucleic acid detection. The study utilized real‐time quantitative fluorescent PCR to detect SARS‐CoV‐2 and evaluate the preservation effect and stability of SARS‐CoV‐2 viral storage solution under various conditions, including different guanidinium salts, brands, and storage conditions.

**Results:**

All brands of inactivated virus preservation solutions demonstrated effective preservation and stability. However, 0.5 mol/L guanidine hydrochloride and guanidine isothiocyanate solutions exhibited poor antiseptic effects. Additionally, refrigerated storage showed better preservation compared to room temperature storage.

**Conclusions:**

We recommend using inactivated virus collection solution to preserve and transport samples and testing preferably within 6 hours to reduce false negatives of NAT results.

## Graphical abstract

We found that the fire‐extinguishing sampling tube (containing guanidine salt) had different effects on the preservation and detection of the virus due to different components, so it was necessary to verify the virus preservation solution.

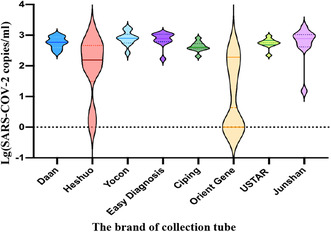

## 1 INTRODUCTION

Coronavirus disease (COVID‐19) caused by severe acute respiratory syndrome coronavirus type 2 (SARS‐CoV‐2) has become a global epidemic.^1^ As of 2 July 2023, over 767 million confirmed cases and over 6.9 million deaths have been reported globally.^2^ Globally, over 885,000 new cases and over 4900 deaths were reported in the last 28 days (5 June to 2 July 2023).^2^ SARS‐CoV‐2 is composed of RNA and protein and has a single‐stranded sense RNA genome.^3^, ^4^ SARS‐CoV‐2 infiltrates host cells by utilizing the angiotensin‐converting enzyme 2 (ACE2) receptor, which is positioned on the luminal surface of epithelial cells,^5^ thus the infection can be confirmed by detecting SARS‐CoV‐2 RNA through collecting human respiratory epithelial cells. Quantitative real‐time reverse transcription‐polymerase chain reaction (qRT‐PCR) is recommended by WHO as a standard confirmation method for SARS‐CoV‐2 detection,^6^, ^7^, ^8^ and specimen collection and nucleic acid testing (NAT) at key sites have become a commonly used clinical option.^9^ Viral NAT involves several steps including sampling, transportation, extraction, and real‐time quantitative polymerase chain reaction (RT‐qPCR) to obtain the final results. The quality of NAT relies on obtaining accurate and timely results.^10^

Virus preservation solution is a medium used to protect and preserve viruses for subsequent tests. There are two types of virus preservation solutions: noninactivated and inactivated. The noninactivated preservation solution mainly consists of Hank's buffer without lysis solution, which helps enhance the survival and stability of the virus during infection. Additionally, ingredients such as gentamicin, antifungal drugs, cryoprotectants, amino acids, bovine serum albumin, and HEPES are usually added to prevent the viral protein shell from easily decomposing and to maintain the virus's activity across a wide temperature range.^11^ Inactivated preservation solution is a modified nucleic acid extraction and lysis preservation solution. This solution contains a high concentration of lytic salts, which enables rapid and efficient lysis of viral proteins in the test sample. Additionally, it contains an RNase inhibitor that effectively protects the viral nucleic acid from degradation. The presence of this solution not only prevents the operator from secondary infection, but also ensures the preservation of the viral nucleic acid.^12^ Inactivated virus preservation solution (IA) usually contains solvent‐promoting (e.g., urea, guanidine, monovalent anion with low charge density, etc.), ribonuclease inhibitors (e.g., diethylpyrocarbonate [DEPC], guanidine isothiocyanate, oxovanadate ribonucleoside complex, SDS, and urea, etc.) and buffer solution, so guanidine salt, alkaline ethylene glycol, EDTA, Tris buffer solution, and sodium citrate can be added.^13^, ^14^ IA allows long‐term transportation and storage at room temperature instead of cold chain transportation, which can save the costs of storage and transportation of virus samples. The use of IA as a medium for virus preservation and transportation can effectively reduce the infection of medical staff.^15^ If the stability and effectiveness of virus preservation solution are poor, it will cause the degradation of viral RNA and false negative results.

Currently, there are numerous brands of virus collection tubes available in China. However, it is important to note that these collection tubes and virus preservation solutions are not regulated as medical devices. Additionally, the quality control requirements for virus preservation solutions may vary among manufacturers. Surprisingly, there is a lack of studies on the effect of preservation solutions on virus preservation or the specific characteristics of virus preservation solutions in NAT research. The storage condition for commercial sample tubes is 1 year at room temperature, but the storage time after sampling is not specified. Additionally, it is important to note that due to distance and operator limitations, the virus sample tubes may be placed for more than 6 h before detection. The preservation stability of virus sampling tubes at room temperature and in cold storage is a significant area of study. This research aims to investigate how the tubes maintain their quality over time. Understanding the stability of these tubes is crucial to ensure their reliability and effectiveness in virus sampling. Additionally, this research allows us to determine the appropriate storage conditions for the tubes, which is essential for preserving the integrity of collected samples. The research findings will offer users a solid research foundation, providing valuable insights into the selection and preservation of viral preservation solution ingredients and brands.

## 2 MATERIALS AND METHODS

### 2.1 Materials

#### 2.1.1 Reagents

The nucleic acid extraction and purification kit (Lot: 2111083, Magnetic beads method; Wuhan Easy Diagnosis) and the SARS‐CoV‐2 nucleic acid detection kit (Lot: 211107, fluorescence PCR method; Wuhan Easy Diagnosis) were used. Additionally, the SARS‐CoV‐2 pseudovirus (Lot: 2021006, with a mean concentration of 1.73E+4 copies/mL; Guangzhou BDS) was included in the experiment. Commercial virus preservation solutions were tested in two groups: the inactivated group (IA) and the noninactivated group (NIA). The inactivated group included products from the following companies: Guangzhou Daan Gene (Lot: 2021009), Hangzhou Heshuo Biological Technology (Lot: 20210911), Yocon Biological Technology (Lot: 26210815), Hangzhou Ciping Medical Instrument (Lot: 26210815), Wuhan Easy Diagnosis Biomedicine (Lot: 210202‐05M), Zhejiang Orient Gene Biotech (Lot: S2101051), USTAR Biotechnologies (Lot: 20210820), and Zhejiang Junshan Gene Science (Lot: QY21K01). The noninactivated group included products from Guangzhou Daan Gene (Lot: 2021004), Hangzhou Heshuo Biological Technology (Lot: 20210911), Yocon Biological Technology (Beijing) (Lot: 01210215), Hangzhou Ciping Medical Instrument (Lot: 202108004), and Wuhan Easy Diagnosis Biomedicine (Lot: 210101‐02).

#### 2.1.2 Storage conditions for sample tubes

The storage conditions and validity period of commercial sample tubes containing viral storage solution are 2–40°C storage for 12 months. The State Council of China's regulations on the storage of virus sample tubes^16^:The specimens collected using the sample tube containing guanidine salt should be transported and stored in accordance with the storage conditions and time requirements specified in the sample tube instructions. If the specimens cannot be detected within 24 h, they should be stored at or below −70°C. If −70°C storage conditions are not available, they can be temporarily stored in a −20°C refrigerator.

#### 2.1.3 Instruments and consumables

The automatic fluorescent PCR analyzer used in this study was the Cobas Z480 (SN: 54339; Roche), while the automatic nucleic acid extraction was performed using the NAE‐96 system from Easy Diagnosis. The experiments were conducted in a biological safety cabinet from Heal Force and a refrigerated centrifuge from Thermo Fisher Scientific, with an oscillator from KylinBell used for mixing. The pipettes used were from Eppendorf, and the tips used were 10, 200, and 1000 μL needle tips with filters from AXYGEN. The LightCycler 480 Multiwell Plate 96 in white (Lot: 21078520; Roche) was also utilized.

### 2.2 Methods

#### 2.2.1 Sample preparation

The self‐prepared inactivated virus preservation solution group (self‐prepared group): Guanidine isothiocyanate (powder, Lot: MB3010‐1; Meilun Bio). Guanidine hydrochloride (powder, Lot: A610242‐0500; Sangong Biology). Sterile normal saline water for preparation and dilution of virus preservation solution (Lot: BL510B; biosharp). the glass tube for configuring sample preservation solution (Lot: DK016110; Dink, China). Prior to the experiment, the glass tube, sterile normal saline water, and experimental consumables underwent autoclaving. Additionally, guanidine isothiocyanate and guanidine hydrochloride were prepared and transformed into guanidinium isothiocyanate(GITC)and Guanidine hydrochloride (GuHCl) solutions with concentrations of 0.5 mol/L, 1.0 mol/L, 2.0 mol/L, 3.0 mol/L, and 4.0 mol/L. These solutions were stored at room temperature (22 ± 2°C) and in a refrigerated environment (6 ± 2°C).

#### 2.2.2 Simulation of positive samples of novel coronavirus

The negative samples (*n* = 20) were collected and centrifuged at 1006 g for 10 min. Epithelial cells were obtained and added into self‐made GITC and GuHCl virus storage tubes with 20 equal parts of high‐concentration epithelial cell suspension, each 50 μL, and 140 μL of the SARS‐CoV‐2 pseudovirus. The mixture was then diluted with normal saline to a total volume of 3 mL, mixed evenly, stored at room temperature, and refrigerated for 60 days, respectively. The concentration of simulated SARS‐CoV‐2 positive samples was 8.07 E+2 copies/mL after the initial addition.

#### 2.2.3 Real‐time fluorescent quantitative polymerase chain reaction

Nucleic acid testing (NAT) was conducted on samples stored for various time intervals ranging from 0 h to 2 months (0, 2, 4, 6, 12, 24, 48, 72 hours, 1 week, and 2 months, respectively). The testing method employed multiplex PCR‐fluorescence probe technology and targeted the 2019‐nCoV ORF1ab and N genes. A dual‐target gene detection primer probe was designed for the RT‐PCR reaction system. Two target genes, including open reading frame 1ab (ORF1ab) and nucleocapsid protein (N), were simultaneously amplified and tested during the real‐time RT‐PCR assay. Target 1 (ORF1ab): forward primer GGTATGTGGAAAGGTTATGGCTGTA; reverse primer ACGATTGTGCATCAGCTGACTG; and the probe 5′‐VIC‐CCCCAGCGCTTCAGCGTTCTTC‐BHQ1‐3′. Target 2 (N): forward primer GATTACAAACATTGGCCGCAAA; reverse primer TGCCAATGCGCGACATTC; and the probe 5′‐FAM‐ATCAACTCCGCGAACCCATGC‐TAMRA‐3′. The results were evaluated by analyzing the threshold cycle (Ct) value of each channel. Additionally, the kit included the human housekeeping gene RNaseP as an internal standard to monitor the sampling, extraction, sample addition, and amplification processes. Prior to use, all reagents were thawed, mixed thoroughly, and centrifuged at 3000 rpm for a few seconds. RT‐qPCR reaction system referred to 20 μL PCR reaction mixture, 5 μL RNA of samples to be tested. Reaction conditions referred to reverse transcription at 50°C for 15 min, predenaturation at 95°C for 30 s, 95°C for 3 s and 60°C for 40 s (40 cycles).

#### 2.2.4 Analysis of results

The specific genes detected by Easy Diagnosis Biomedicine amplification reagent were ORF1ab and N gene. S1, S2, S3, S4, and S5 standards were prepared with concentrations of 1.73E+4, 8.65E+3, 4.33E+3, 2.16E+3, and 1.08E+3 copies/mL, respectively. These standards were created by doubling dilution with BDS Biological Technology peak value sample (concentration of 1.73E+4 copies/mL) four times. The diluent used was sterile normal saline water. A standard curve (*R* ^2^ ≥ 0.95) was plotted using the above 5 points, forming a standard curve that could be used to calculate the concentration of SARS‐CoV‐2 nucleic acid in the sample. In this study, the ORF1ab gene was selected as the target gene to determine the virus concentration. The logarithm of the virus concentration was used as the final data for analysis and plotting. The calculation formula was as follows:

$$N=\mathrm{lg}\left(\text{SARS}‐\mathrm{CoV}‐2\;\text{copies}/\mathrm{mL}\right).$$

### 2.3 Statistical analysis

To compare the difference between inactivated and noninactivated virus preservation solutions of the same brand at different preservation times, two related sample tests (Wilcoxon) were conducted. The preservation difference between different brands in the noninactivated virus preservation solution was compared using K Related samples tests in Nonparametric Tests. Additionally, the Wilcoxon test was performed to compare the efficacy and stability of the same concentration of GITC and GuHCl groups for refrigerated and room‐temperature storage of the SARS‐CoV‐2. The Friedman test was performed to compare the stability of SARS‐CoV‐2 preservation among different concentrations of GuHCl solution, different concentrations of guanidine isothiocyanate solution, and different brands of virus preservation solution. The Mann–Whitney *U* test was used to assess the numerical differences in stability between inactivated and noninactivated virus preservation solutions. The above statistical analyses were conducted using SPSS, Version 26.0.

## 3 RESULTS

### 3.1 Comparison of preservation effects of inactivated and noninactivated virus preservation solutions from the same brand under refrigerated temperatures

**FIGURE 1:**
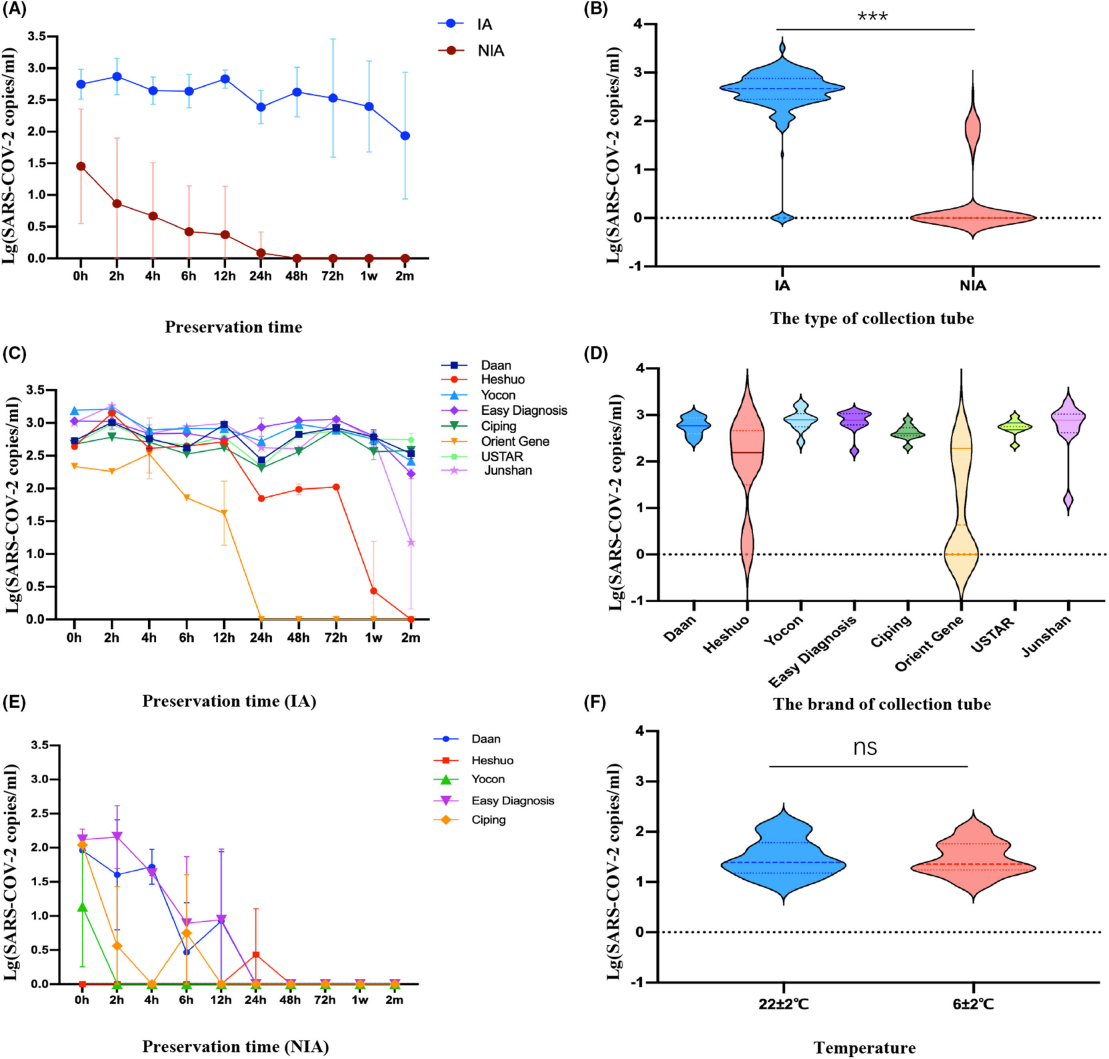
Comparison of preservation effect and stability in inactivated and noninactivated preservation solution. (A, B) Comparison between IA and NIA; (C, D) Comparison of IA among different brands; (E) Comparison of NIA among different brands; (F) Comparison of storage under different storage temperatures in self‐prepared group. *p < 0.05, **p < 0.01,***p < 0.001.

We stored two aliquots of virus preservation solution, of the same volume, from multiple brands of inactivated (IA) and noninactivated (NIA) solutions for different periods of time, as described in Section 2.2.1. Subsequently, we conducted real‐time PCR reactions to detect the concentration of SARS‐CoV‐2 nucleic acid and analyzed the results. Table 1, Figure 1A,B clearly demonstrate that the preservation stability of IA is superior to that of NIA (*t* = 39.07, *p <* 0.001). According to Figure 1C,D, it can be observed that the stability of Orient Gene and Heshuo IA was relatively low, as indicated by a clear downward trend in their curves. Notably, the long‐term storage stability of NIA in our experiment was poor, as the virus could not be detected after 2 h of storage in 2 out of 5 brands (Figure 1E).On the other hand, there was no significant difference observed among the five brands of NIA virus preservation solution (*χ* ^2^ = 5.806, *p* = 0.214).

**TABLE 1:** Comparison of preservation effects of the same brand of inactivated and noninactivated virus preservation solution at refrigerated temperatures.

Brand	Type	0 h	2 h	4 h	6 h	12 h	24 h	48 h	72 h	1 week	2 months	*p* (*Z*)
Daan gene	IA	2.50	2.06	2.57	2.04	2.74	2.78	2.42	2.65	2.57	2.58	0.007 (−2.701)
	NIA	1.99	2.26	1.56	0.00	1.88	0.00	0.00	0.00	0.00	0.00	
Heshuo	IA	2.49	2.89	2.40	2.45	2.66	2.25	1.79	0.00	2.44	0.00	0.012 (−2.521)
	NIA	0.00	0.00	0.00	0.00	0.00	1.73	0.00	0.00	0.00	0.00	
Yocon	IA	2.83	3.11	2.66	2.69	2.91	2.53	2.54	2.77	2.82	2.73	0.005 (−2.803)
	NIA	1.92	0.00	0.00	0.00	0.00	1.75	0.00	0.00	0.00	0.00	
Ciping	IA	2.58	2.58	2.46	2.44	2.76	2.54	2.38	2.67	2.57	3.16	0.005 (−2.803)
	NIA	2.02	0.00	0.00	1.76	0.00	0.00	0.00	0.00	0.00	0.00	
Easy diagnosis	IA	2.97	3.02	2.97	3.00	3.05	3.09	2.99	3.70	2.90	2.82	0.005 (−2.803)
	NIA	2.26	2.34	1.67	0.00	1.88	0.00	0.00	0.00	0.00	0.00	

### 3.2 Comparison of the NAT ability of different brands of inactivated virus preservation solution at different temperatures

Eight types of inactivated virus preservation solution were stored at room temperature (22 ± 2°C) and refrigerated (6 ± 2°C). Nucleic acid extraction and viral nucleic acid detection were performed at various time intervals ranging from 0 h to 2 months. We compared the preservation effect of the same brand of inactivated virus preservation solution at room temperature and in a refrigerated environment. The results showed that there was no significant difference in the preservation effect between different brands of virus preservation solutions at room temperature and in a refrigerated environment(*p* all >0.05, see Table 2 and Figure 1F), except for Easy Diagnosis preservation solution, which exhibited better preservation effect in the refrigerated environment (*Z* = ‐2.295, *p* = 0.022, see Table 2).

**TABLE 2:** Comparison of the preservation effect of the same brand of inactivated virus preservation solution at room temperature (22 ± 2°C) and refrigerated temperatures (6 ± 2°C).

Brand	Type	0 h	2 h	4 h	6 h	12 h	24 h	48 h	72 h	1 week	2 months	*p* (*Z*)
Daan gene	22 ± 2°C	2.66	3.13	2.70	2.61	2.96	2.46	2.67	3.09	2.57	2.58	0.333 (−0.968)
	6 ± 2°C	2.50	2.06	2.57	2.04	2.74	2.78	2.42	2.65	2.90	2.94	
Heshuo	22 ± 2°C	2.66	3.30	2.40	2.61	2.69	1.78	1.85	2.31	1.52	0.00	0.484 (−0.700)
	6 ± 2°C	2.49	2.89	2.40	2.45	2.66	2.25	1.79	0.00	2.44	0.00	
Yocon	22 ± 2°C	3.28	2.73	2.91	2.89	2.91	2.72	2.86	3.09	2.78	3.03	0.109 (−1.601)
	6 ± 2°C	2.83	3.11	2.66	2.69	2.91	2.53	2.54	2.77	2.82	2.73	
Ciping	22 ± 2°C	2.77	3.05	2.72	2.51	2.64	2.50	2.44	3.15	2.74	2.76	0.139 (−1.478)
	6 ± 2°C	2.58	2.58	2.46	2.44	2.76	2.54	2.38	2.67	2.57	3.16	
Easy diagnosis	22 ± 2°C	3.00	3.01	2.86	2.83	2.72	2.87	2.92	3.06	2.93	2.58	0.022 (−2.295)
	6 ± 2°C	2.97	3.02	2.97	3.00	3.05	3.09	2.99	3.70	2.90	2.82	
Orient gene	22 ± 2°C	2.27	2.07	1.54	1.82	0.00	0.00	0.00	0.00	0.00	0.00	0.116 (−1.572)
	6 ± 2°C	2.80	2.33	1.84	1.50	2.09	1.48	0.00	0.00	0.00	0.00	
USTAR	22 ± 2°C	2.78	1.95	2.78	2.66	2.77	2.52	2.69	3.02	2.77	3.19	0.169 (−1.376)
	6 ± 2°C	2.81	2.54	2.48	0.00	2.99	2.40	2.60	1.78	2.59	2.86	
Gene science	22 ± 2°C	3.03	3.32	2.81	2.91	2.97	2.73	2.52	3.16	2.92	1.89	0.203 (−1.274)
	6 ± 2°C	2.51	2.97	2.44	2.60	2.63	2.83	2.55	3.07	2.88	2.61	

### 3.3 Comparison of preservation effects of the self‐prepared virus preservation solution

**FIGURE 2:**
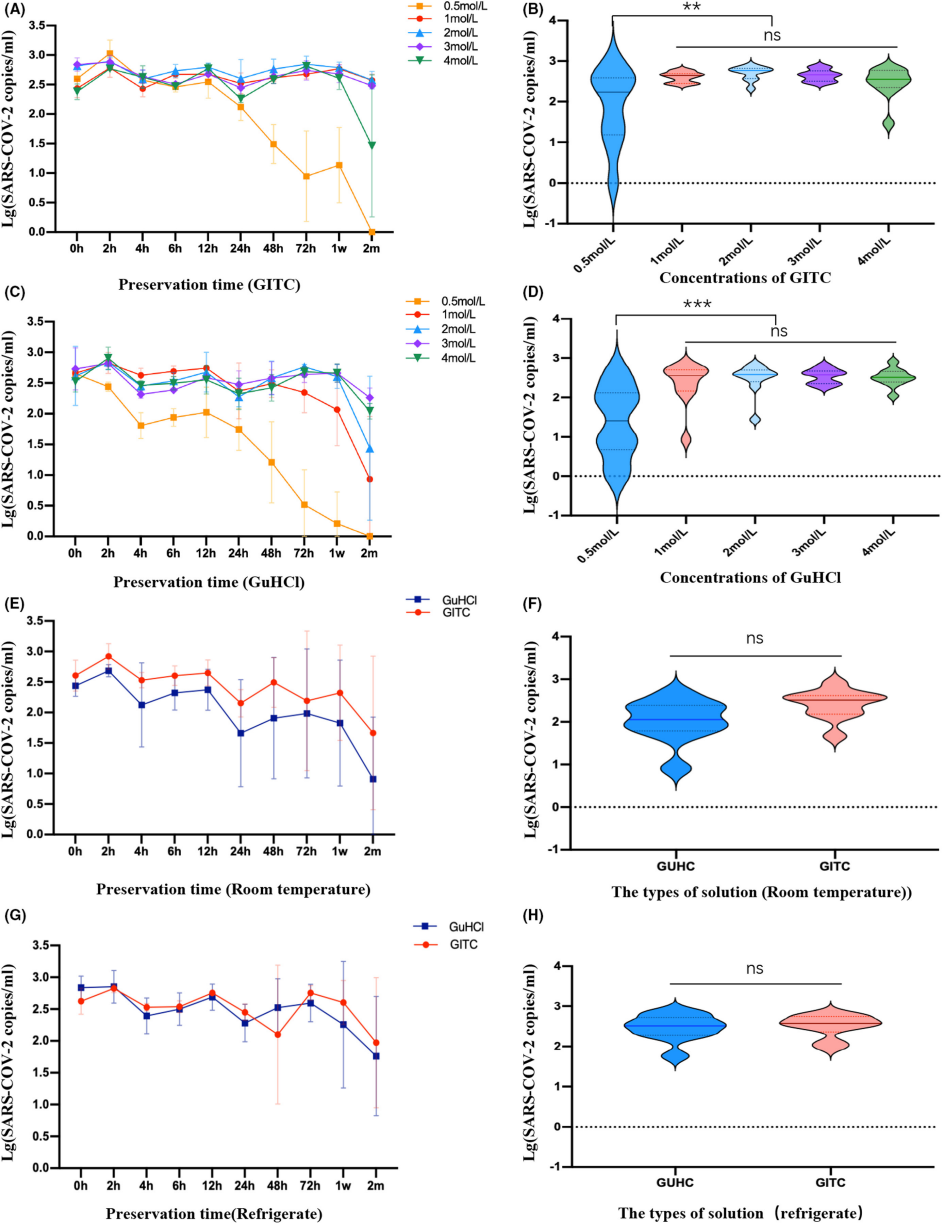
Comparison of preservation effect and stability in different concentrations of GuHCl and GIHC. (A, B) Comparison of GuHCl solutions at different concentrations; (C, D) Comparison of GuHCl at different concentrations; (E, F) Comparison of GuHCl and GIHC at room temperature. (G, H) Comparison of GuHCl and GIHC at refrigerated condition. *p < 0.01, **p < 0.01,***p < 0.001.

In our study, we observed that the refrigerated preservation effect in the self‐prepared virus preservation solution group was significantly better than that at room temperature (refrigeration: 5.38 [±1.61] E+02 copies/mL; room temperature: 4.34 [±1.95] E+2 copies/mL, *Z* = −2.701, *p* < 0.05). Additionally, we noticed a clear downward trend in the guanidine content of 0.5 mol/L in both GITC and GuHCl groups (Figure 2A,C). Compared to other guanidine concentration groups, viral nucleic acid degradation of 0.5 mol/L guanidine content was more pronounced(*p* all >0.01; Figure 2B,D).

The degradation rates of viral nucleic acid in 0.5, 1.0, 2.0, 3.0, and 4.0 mol/L GITl solution at 48 h were 45.2%, 4.78%, 18.9%, 15.0%, and 5.00%, respectively. After being placed in 0.5 mol/L GITC solution for 48 h, the NAT kit could not detect the virus, and all the viruses were degraded after being placed for 60 days (Figure 2). The concentration of viral nucleic acid in GITC solution of other concentrations did not decrease significantly after 60 days. The degradation rates of viral nucleic acid in 0.5, 1.0, 2.0, 3.0, and 4.0 mol/L GuHCl solution at 48 h were 100%, 3.34%, 10.2%, 11.7%, and 8.49%, respectively. As shown in Figure 2, although there was no significant difference in viral nucleic acid concentration between guanidine isothiocyanate and guanidine hydrochloride virus storage solution at room temperature and in cold storage, there was a better correlation between the viral nucleic acid detection curves of guanidine isothiocyanate and guanidine hydrochloride virus storage solution in cold storage, as shown in Figure 2.

## 4 DISCUSSION

As a medium for transporting and preserving viruses, the virus preservation solution plays a crucial role in ensuring the accuracy of test results. Currently, there are numerous brands of virus preservation solutions available, each with different ingredients. However, there is currently no standardized unified standard for these solutions in China. The virus preservation solution plays a vital role in ensuring stable preservation and minimizing the risk of virus transmission. If the solution does not effectively preserve the nucleic acid, there is a higher chance of obtaining false negative results. The World Health Organization (WHO) recommends using qRT‐PCR as the standard method for confirming SARS‐CoV‐2 detection.^6^, ^7^, ^8^ Several studies have examined the extent of degradation of SARS‐CoV‐2 viral nucleic acid by analyzing the presence or absence of tailing and diffusion of the bands in the electropherogram.^17^ In this study, the RT‐qPCR technique was employed to determine viral nucleic acid concentration as it provides a more intuitive comparison of the preservation stability of different viral storage solutions. This is because electrophoretic technology is not suitable for quantitatively detecting nucleic acid concentration. In evaluating the preservation effect of IA and NIA, we observed that IA demonstrated higher stability in preserving SARS‐CoV‐2. This could potentially be attributed to the presence of RNase inhibitors, which protect the viral nucleic acid from degradation. Among all IA used in this study, except for Heshuo and Orient gene brands, the NAT by other brands of virus preservation solution within 12 h had little effect on the results. Based on the analysis of Figures 1A and 2A,C, it is evident that a significant difference in measurements exists when the virus concentration is below 2(lg100 copies/mL). This discrepancy suggests that the concentration is in proximity to the gray area of fluorescence quantitative detection.

In this study, we prepared guanidinium salt virus preservation solution with different concentrations to investigate their preservation stability against SARS‐CoV‐2, finding that the use of GITC preservation solution could reduce the probability of false negative results.^18^ A previous study on rapid antigen detection and inactivation of SARS‐CoV‐2^19^ also recommended chemical inactivation (i.e., IA) for subsequent testing. Previous studies have reported that guanidinium salts can create a high‐salt environment, offering protection to nucleic acid molecules and reducing their degradation. These salts also have the ability to inhibit RNase.^20^ Referring to GITC for chemical lysis of viruses,^13^, ^18^, ^21^ we utilized guanidinium hydrochloride and GITC as virus preservation buffers and both of them demonstrated good preservation stability when used at appropriate concentrations. A concentration of 0.5 mol is not recommended for guanidine content as a viral preservation solution for NAT. GuHCl is a powerful ionic protein denaturant, while GITC has an even stronger ability to denature proteins. A concentration of 4.0 mol/L of GITC is the preferred denaturant for extracting RNA from RNase‐rich samples.^22^ Previous research has demonstrated that GIHCl exhibits a superior ability to effectively eradicate viruses and inhibit RNase. As a result, it is commonly included in nucleic acid extraction buffers.^23^ For example, TRIzol reagent was a single‐phase solution containing phenol and GITC, which is often used for deproteinization to extract RNA.^24^

Since the samples were unable to meet the required refrigeration conditions of 2–8°C prior to testing, this study aimed to compare the effects of storage under room temperature (20–24°C) and refrigeration (2–8°C) on the preservation solution for the virus. The results showed no significant difference between the virus preservation solution stored at room temperature and refrigeration. This suggests that the buffer solution and other components added to the ready‐made IA have a better ability to resist interference from temperature, pH changes, and temperature fluctuations. The aforementioned results align with a previous study that utilized Innomed VTM001 virus collection tubes to compare the impact of two distinct storage temperatures (i.e., 22 ± 2°C and 6 ± 2°C) on 30 positive samples by RT‐PCR analysis for SARS‐CoV‐2, finding no false negative results within a span of 6 days for all samples.^25^ It has been proven that storing self‐prepared virus preservation solution in cold storage can make the preservation of viruses more difficult to degrade over a longer period of time. This finding is supported by the interim guidance on COVID‐19 specimen processing and transportation provided by the Center for Disease Control in the U.S.,^26^ healthcare professionals should store respiratory specimens at 2–8°C for up to 72 h after collection, or −70°C or lower if testing or transportation was delayed. Due to the composition of self‐prepared virus preservation solution and its sensitivity to temperature change, commercial ready‐made IA had better virus preservation effect, in which different brands exhibit varying levels of stability in preserving viruses. A potential limitation of this study is that the stability of noninactivated virus sampling solution may be affected by the length of pseudoviral fragments and the degradation of virus‐specific RNAs caused by RNases in human epithelial cells. However, the advantage of the study of pseudovirus particles is that it can make a standardized curve to accurately quantify the degree of degradation of the SARS‐CoV‐2 pseudovirus particles so that the degree of degradation can be expressed in a digital form.

In conclusion, we highly recommended to use inactivated virus collection solution to preserve and transport samples in order to reduce the false negative in NAT results, improve the accuracy of NAT, ensure virus preservation stability, and minimize the risk of virus transmission. Additionally, refrigerated preservation is more suitable for identifying new coronavirus nucleic acid. If it is not possible to perform performance verification of the virus preservation solution before the experiment, NAT should be conducted in the laboratory as soon as possible, preferably within 6 h.

## AUTHOR CONTRIBUTIONS

Xiuzhi Duan, Letao Huang, and Zhihua Tao contributed to bibliographic research, drafting of the paper, critical revision, and final approval of the submitted version. Xuchu Wang, Yiyi Xie, and Weiwei Liu contributed to critical revision and final approval of the submitted version. Ying Ping and Pan Yu contributed to study conception and design, critical revision, and final approval of the submitted version. All authors read and approved the final manuscript.

## CONFLICT OF INTEREST STATEMENT

None.

## CONSENT FOR PUBLICATION

All patients were informed and agree to publish.

## Data Availability

The data that support the findings of this study are available from the corresponding author upon reasonable request.
